# Encoding of Tactile Stimuli by Mechanoreceptors and Interneurons of the Medicinal Leech

**DOI:** 10.3389/fphys.2016.00506

**Published:** 2016-10-28

**Authors:** Jutta Kretzberg, Friederice Pirschel, Elham Fathiazar, Gerrit Hilgen

**Affiliations:** ^1^Computational Neuroscience, Department of Neuroscience, University of OldenburgOldenburg, Germany; ^2^Cluster of Excellence Hearing4all, University of OldenburgOldenburg, Germany; ^3^Department of Organismal Biology and Anatomy, University of ChicagoChicago, IL, USA; ^4^Faculty of Medical Sciences, Institute of Neuroscience, Newcastle UniversityNewcastle upon Tyne, UK

**Keywords:** mechanoreception, somatosensory system, touch, pressure, skin stimulation, voltage sensitive dye, local bend, hirudo

## Abstract

For many animals processing of tactile information is a crucial task in behavioral contexts like exploration, foraging, and stimulus avoidance. The leech, having infrequent access to food, developed an energy efficient reaction to tactile stimuli, avoiding unnecessary muscle movements: The local bend behavior moves only a small part of the body wall away from an object touching the skin, while the rest of the animal remains stationary. Amazingly, the precision of this localized behavioral response is similar to the spatial discrimination threshold of the human fingertip, although the leech skin is innervated by an order of magnitude fewer mechanoreceptors and each midbody ganglion contains only 400 individually identified neurons in total. Prior studies suggested that this behavior is controlled by a three-layered feed-forward network, consisting of four mechanoreceptors (P cells), approximately 20 interneurons and 10 individually characterized motor neurons, all of which encode tactile stimulus location by overlapping, symmetrical tuning curves. Additionally, encoding of mechanical force was attributed to three types of mechanoreceptors reacting to distinct intensity ranges: T cells for touch, P cells for pressure, and N cells for strong, noxious skin stimulation. In this study, we provide evidences that tactile stimulus encoding in the leech is more complex than previously thought. Combined electrophysiological, anatomical, and voltage sensitive dye approaches indicate that P and T cells both play a major role in tactile information processing resulting in local bending. Our results indicate that tactile encoding neither relies on distinct force intensity ranges of different cell types, nor location encoding is restricted to spike count tuning. Instead, we propose that P and T cells form a mixed type population, which simultaneously employs temporal response features and spike counts for multiplexed encoding of touch location and force intensity. This hypothesis is supported by our finding that previously identified local bend interneurons receive input from both P and T cells. Some of these interneurons seem to integrate mechanoreceptor inputs, while others appear to use temporal response cues, presumably acting as coincidence detectors. Further voltage sensitive dye studies can test these hypotheses how a tiny nervous system performs highly precise stimulus processing.

## Introduction

A simple neuronal system produces a basic behavior with a surprisingly high precision: The leech bends away locally from a light touch (Stuart, 1970; Kristan, 1982; Lockery and Sejnowski, 1992; Lewis and Kristan, 1998a; Zoccolan et al., 2002; Baca et al., 2005; Thomson and Kristan, 2006) with a spatial precision of approximately 1 mm (Baca et al., 2005); similar to that of the human fingertip (Johnson, 2001). The different leech mechanoreceptor types show similar spiking patterns to primate and human mechanoreceptor types (Lewis and Kristan, 1998b; Baca et al., 2005; Johansson and Flanagan, 2009; Smith and Lewin, 2009), and mechanoreceptor responses were shown to depend on common stimulus properties like touch location, mechanical force, duration, and speed (leech: Carlton and McVean, 1995; Zoccolan et al., 2002; Baca et al., 2005; Pirschel and Kretzberg, 2016; primate reviews: Johansson and Flanagan, 2009; Abraira and Ginty, 2013; Saal and Bensmaia, 2014). However, the number of mechanoreceptor cells in the leech skin is an order of magnitude lower than in the human fingertip, which is innervated by more than 200 mechanoreceptors per cm^2^ (Vallbo and Johansson, 1984). Nevertheless, the complex innervation structure of the leech skin enables the highly accurate and reproducible local bend response to avoid being stimulated with minimal muscle movement. Hence, the small and simple neuronal system of the leech raises a fundamental computational question on sensory processing: How can such a precise behavior be performed with a nervous system consisting of so few cells?

The leech nervous system is a rigorously segmented, highly repetitive ventral nerve cord with one ganglion per segment. Each ganglion contains about 400 neurons of approximately 200 types (Kristan et al., 2005). The leech local bend behavior was suggested to be controlled by a three-layered feed-forward network consisting of mechanoreceptors, interneurons, and motor neurons (Kristan, 1982; Lockery and Kristan, 1990b; Lewis and Kristan, 1998a; Kristan et al., 2005), which are found in each segment of the animal.

**Figure 1:**
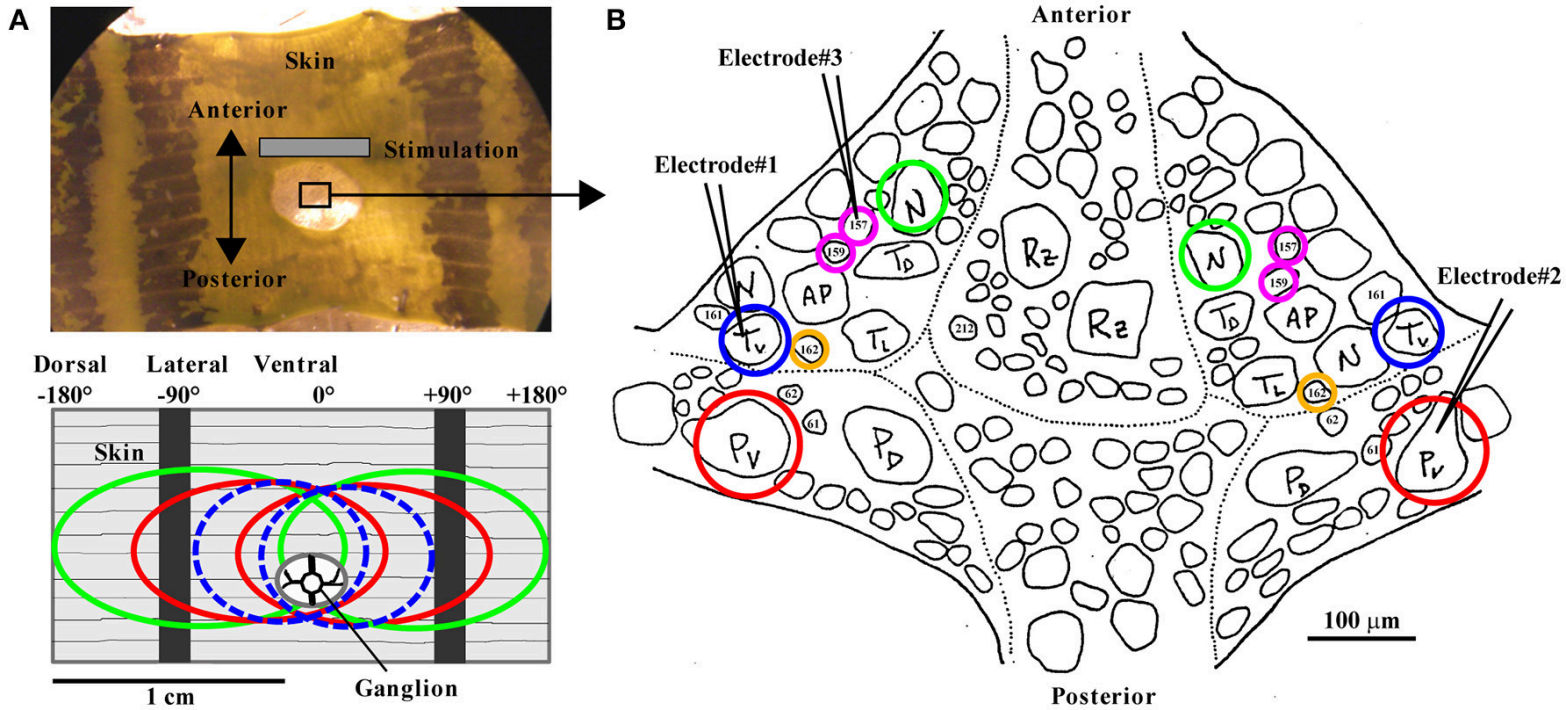
**The body-wall preparation with receptive fields of mechanoreceptors and standard ganglion map**. (**A**, top) Photograph of the body-wall preparation with the ganglion (sketch in **B**), which is pulled slightly posterior for better access through a hole in the skin. The gray bar indicates the main area used for tactile stimulation. The center of the preparation between the two dark stripes on the skin, called the ventral midline, was defined as 0°. The skin was touched at the third annulus of segment 10, identified by the sensilla positions. Touch locations to the left were denoted as negative numbers of degrees (left end of the preparation: −180°) and to the right as positive numbers (right end: +180°). The black stripes are located approximately at −90° and +90°. (**A**, bottom) Receptive fields of all mechanoreceptors responding to tactile stimulation at the ventral midline are shown in a sketch of the body wall preparation: Left and right T_v_ cells (dashed blue ovals), left and right P_v_ cells (red), and left and right N cells (green). **(B)** Sketch of the leech ganglion with cell body positions of bilateral mechanoreceptor pairs of P_v_ (red), T_v_ (blue), N (green), corresponding to the receptive fields shown in **(A)**, and of interneurons 157, 159 (magenta), 162 (yellow). Electrodes (symbolized by black pointed angles) were used for intracellular recordings of up to 3 neurons (combinations of T_v_, P_v_, N, 157, 159, and 162; the electrode positions shown here refer to the data shown in Figures 5A,B), while the skin was stimulated mechanically (see section Methods).

The input layer of the local bend network consists of mechanoreceptors. The three types of leech mechanoreceptors were classically associated with tactile stimuli of distinct intensities, resulting in the names of these neurons: T cells for light touch, P cells for stronger pressure, and N cells for noxious, very strong squeeze (Nicholls and Baylor, 1968). In computational terms, these distinct functions refer to a labeled line code. Moreover, spike patterns in response to tactile skin stimulation differ characteristically between receptor types. T cells produce transient, fast adapting responses to stimulus on- and offset, while P cells respond with sustained, regular spiking throughout the stimulation and N cells produce only few spikes separated by long interspike intervals (Nicholls and Baylor, 1968; Carlton and McVean, 1995; Lewis and Kristan, 1998b; Pirschel and Kretzberg, 2016). In each segment the total population of mechanoreceptors consists of only 14 cells (6 T, 4 P, 4 N) innervating the skin at different depths with their processes (Blackshaw, 1981; Blackshaw et al., 1982) and sending information about tactile stimuli toward their cell bodies in the segmental ganglion. Skin regions innervated by several cells lead to widely overlapping receptive fields between mechanoreceptors. For example, tactile stimulation applied to the ventral midline cause spike responses in two P, two T, and two N cells (see Figure 1A bottom for a sketch of overlapping receptive fields at ventral midline). In all mechanoreceptors the inhomogeneous distribution of dendritic branches and nerve endings in the skin (Blackshaw, 1981; Blackshaw et al., 1982) cause spatially structured receptive fields. Stimulation close to the most densely innervated receptive field center triggers highest spike counts and shortest spike latencies (Nicholls and Baylor, 1968; Thomson and Kristan, 2006; Pirschel and Kretzberg, 2016).

Since P cell responses were found to influence muscle movements more strongly than T cells, previous studies assumed that P cells elicit the local bend reflex (Kristan, 1982; Lewis and Kristan, 1998b; Zoccolan et al., 2002). Therefore, a prior study aiming at the identification of interneurons involved in the local bend network was limited to neurons responding to presynaptic P cell spikes (Lockery and Kristan, 1990b). Based on a huge number of double recordings this study identified one unpaired and eight paired interneurons to be involved in the local bend network. Most of these interneurons responded with postsynaptic potentials to spikes from all four P cells suggesting very extended receptive fields. Substantial lateral synaptic connections between interneurons were not found in this study except for electrical connections between the pair of interneurons of the same type (Lockery and Kristan, 1990b). In contrast, identified motor neurons were found to be laterally connected. In addition to inhibiting longitudinal muscles, inhibitory motor neurons also suppress excitatory motor neurons, which cause contraction of the antagonistic muscles (Granzow and Kristan, 1986; Lockery and Kristan, 1990a; Kristan et al., 2005; Baca et al., 2008). This antagonistic inhibition leads to a stronger bending movement to one side.

Based on these results, Lewis and Kristan (1998a) developed a computational model of the local bend network. The model consists of three layers of cells with evenly spaced cosine shaped tuning curves, implementing a population vector paradigm as optimal decoding scheme from one layer to the next. Using the spike rates of the four P cells as input, this model predicted the behaviorally observed direction of local bending. While the cell numbers of the input and output layers were fixed to 4 P cells and 10 motor neurons, the number of the much less-known interneurons was varied, revealing that the number of 17 previously identified local bend interneurons (Lockery and Kristan, 1990b) was compatible with the modeled network structure.

Despite the elegant plainness of this model, recent results require to revise the hypothesized structure and computations of the local bend network. Stimulus-estimation studies revealed that latency differences between two P cell responses carry more information about tactile stimulus position on the skin than spike counts (Thomson and Kristan, 2006; Pirschel and Kretzberg, 2016). Hence, temporal response features might play an important role in the network. Moreover, latency differences between T cell pairs allowed an even more exact estimation of stimulus location (Pirschel and Kretzberg, 2016). Therefore, P cell spikes might not be the only relevant input to the network, but the role of T cells—and maybe also N cells—in the network should be reconsidered. Another aspect not covered by the classical local bend model is the fact that the behavioral response to a tactile stimulus depends on a combination of stimulus properties like location, mechanical force intensity and duration (Baca et al., 2005), as well as velocity (Carlton and McVean, 1995). On the mechanoreceptor level, the finding that several response features, including spike count and latency, depend on more than one stimulus property leads to the fundamental question how complex stimuli are encoded by the nervous system. For example, stimulus location and mechanical force intensity influence the neuronal responses of the mechanoreceptor types in an ambiguous way. A mechanoreceptor response with a high spike count and a short response latency could be elicited either by a relatively weak stimulus close to the cell's receptive field center, or by a stronger stimulus at a less preferred position (Pirschel and Kretzberg, 2016). How does the leech distinguish between these stimuli based on such ambiguous responses?

Our hypothesis is that a population of interneurons solves this task by means of multiplexing, simultaneous encoding of different stimulus properties with different response features. The population of T and P cells provides multiplexed information about combinations of e.g., stimulus location and intensity by encoding them simultaneously with temporal and spike count features (Pirschel and Kretzberg, 2016). The first aim of this study is to investigate if interneurons respond specifically to one mechanoreceptor type—indicating a labeled line code, as it was classically assumed for intensity encoding—or if they integrate inputs from multiple receptor types. The second question is, which mechanoreceptor response features determine interneuron responses. Are all of them integrators as it was assumed in the spike rate-based computational network model (Lewis and Kristan, 1998a), or are some of them specialized for temporal processing?

In the first part of this study, responses of all three types of mechanoreceptors (T, P, and N cells) to tactile skin stimulation are revisited. Our recent results (Pirschel and Kretzberg, 2016) are extended by adding N cell responses and the analysis of two-dimensional tuning to combinations of different stimulus locations and intensities. In the second part, intracellular electrophysiology, anatomical studies and voltage sensitive dye recordings are performed as complementary experimental approaches to study interneurons on the next network layer. In particular, we aim to identify interneurons responding to input from P and/or T cells and to find out which mechanoreceptor response features determine their postsynaptic responses. In this way, we try to identify general computational principles of sensory information processing, which are not limited to the leech, but could be implemented also by other sensory systems.

## Methods

### Animals and preparations

All experiments were performed on adult, hermaphrodite medicinal leeches (Hirudo verbana), weighing 1–2 g. According to German regulations, no ethics approval is needed for the work on these invertebrates. Leeches were obtained from Biebertaler Leech Breeding Farm (Biebertal, Germany) and were kept in tanks with Ocean Sea Salt 1:1000 diluted with purified water. Animals were kept at room temperature and anesthetized with ice-cold saline (Muller and Scott, 1981) before and during dissection. Experiments were performed at room temperature.

For the body-wall preparation (Figure 1A; detailed description is given in Pirschel and Kretzberg, 2016), segments 9–11 were dissected and innervations of segment 10 remained unscathed, while the ganglion was accessible through a hole in the skin (Figure 1A). The middle annulus of the 10th segment, which was identified by the location of the sensilla (Blackshaw et al., 1982), was used for the skin stimulation.

Voltage sensitive dye (VSD) experiments (section Voltage Sensitive Dye Experiments and Analysis) and cell fills (section Dye Injection and Cell Labeling) were performed on isolated ganglia dissected from segment 10.

### Tactile stimulation and intracellular electrophysiology

In the skin preparation, the skin was stimulated by the Dual-Mode Lever Arm System (Aurora Scientific, Ontario, Canada, Model 300B; poker tip size: 1 mm^2^; see Baca et al., 2005; Thomson and Kristan, 2006; Pirschel and Kretzberg, 2016). The stimulus was applied as an instantaneous step function of 200 ms length. At stimulus onset, the poker moved down at very high speed, reached the desired pressure within 2 ms, fluctuated slightly for less than 10 ms and stayed at a constant position, until moving up at very high speed again. Poker speed and duration of skin indentation were the same in all experiments. Touch locations were set relative to the ventral midline (set as 0°) of the preparation: Locations to the left are denoted as negative and to the right as positive numbers of degrees (Figure 1A). The stimulus was varied in mechanical force intensity (5–200 mN) and location (−20° to +20°, relative to the ventral midline, in 5° steps) (see Lewis and Kristan, 1998b; Baca et al., 2005; Pirschel and Kretzberg, 2016). Other parameters like shape or indentation depth were not varied. All combinations of stimulus properties force intensity and location were presented 8–15 times in pseudo-randomized order.

While stimulating the skin mechanically, intracellular recordings from one to three cells at the same time were performed with sharp glass micropipettes (resistances between 20 and 40 MΩ) filled with 3 M potassium acetate (for detailed description of the experimental rig, see Pirschel and Kretzberg, 2016). Varied combinations of the three types of mechanosensory cells (T_v_ and P_v_, N) and three types of interneurons (157, 159, and 162) were obtained. Numbers of preparations used for analyses are given in the figure legends. Mechanosensory cell types were easily identifiable based on their electrical properties (Nicholls and Baylor, 1968). In tactily stimulated preparations, the receptive field of recorded mechanoreceptors was confirmed prior to experiments, yielding the standard map shown in Figure 1B. In most ganglia, cell bodies of T_v_ and P_v_ (the mechanoreceptors with ventral receptive fields) were located most laterally. But since in particular T cells sometimes switch their positions, we specify in this manuscript subtypes of T and P cells only for experiments with attached skin. The interneurons (INs) were identified according to the results and descriptions by Lockery and Kristan (1990b).

To physiologically identify synaptic connections, intracellular double recordings of INs and a simultaneous recording of a mechanosensory cell were obtained, while stimulating the mechanosensory cell by constant current pulses of 1.5 nA, lasting 50 ms.

### Dye injection and cell labeling

To study cell morphologies and putative points of contact, interneurons, and mechanosensory cells were filled in isolated ganglia by means of sharp glass electrodes (20–40 MΩ) with 10 mM Alexa-dyes (Alexa Fluor 488/546/633, Invitrogen, Karlsruhe, Germany) and/or 2% Neurobiotin (Vector Labs, Peterborough, UK) solved and backfilled with 200 mM KCl. Cells were iontophotoretically injected either with positive (Neurobiotin) or negative (Alexa) currents (2–4 nA, 500 ms, 1 Hz, 30–60 min). Neurobiotin-filled samples were allowed to settle for 1 h after injection before further processing. All samples were fixated in 4% PFA (Sigma, Munich, Germany) for 1 h and rinsed 6 × 10 min in 0.1 M PBS. Neurobiotin-filled samples were afterwards incubated in 1:1000 Streptavidin DyLight 488 (Vector Labs)/PBS/0.5% Triton-X overnight at 4°C. Samples were rinsed afterwards (6 × 10 min) in PBS and embedded with VectaShield (Vector Labs) on a microscope slide for high resolution microscopy. Fluorescent image acquisition and analysis were performed as previously described (Meyer et al., 2014). Briefly, filled cells were scanned with a Leica TCS SP2 (Leica, Nussloch, Germany) Confocal Microscope with an HCX PL APO 40.0 × 1.25 OIL UV objective to obtain confocal stacks with a voxel dimension of 0.366 × 0.366 × 0.200 μm. The scanned sequential images were trimmed for the desired z-depth and a maximal projection of the images was calculated with ImageJ (NIH, Bethesda, MD). Channel overlay and gentle adjustment of contrast and brightness were done with Photoshop CS3 (Adobe, San Jose, CA). An animation of the confocal stack underlying **Figure 6B** is provided in the Supplemental Material.

### Voltage sensitive dye experiments and analysis

**Figure 2:**
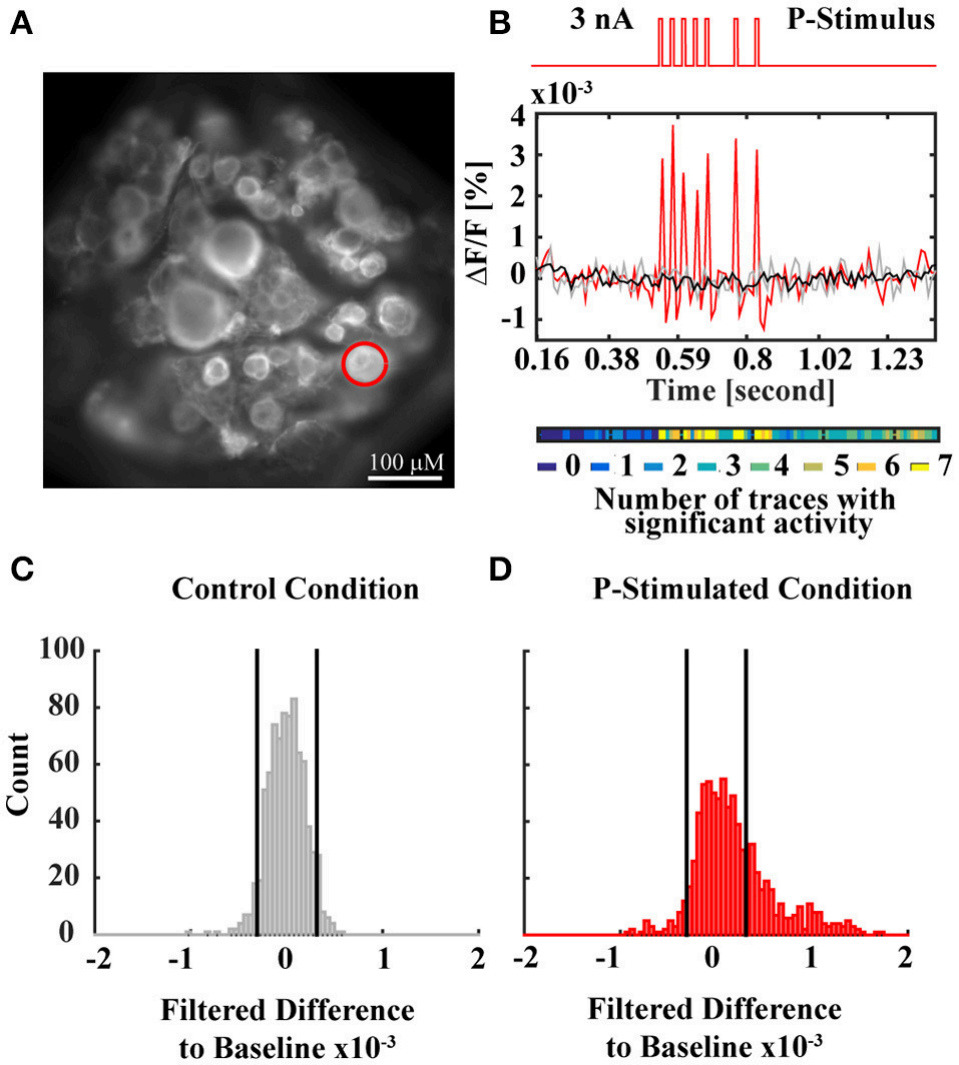
**VSD data analysis method. (A)** Snapshot of a ganglion with full resolution. The red circle indicates the cell body of the P cell, which was stimulated by intracellular current injection into the soma during the experiment. **(B)** Time course of the electrical P cell stimulation, applied through an intracellular electrode (top red trace) and corresponding single VSD response of the P cell (red spikes) in comparison to a single P cell response to control condition (not stimulated, gray) and baseline (averaged P cell responses to 7 traces of control condition, black). The activity map (colored line below) indicates for each recording frame in how many of 7 response traces the activity differed significantly from control condition. **(C)** Histogram of filtered differences between P cell responses to control condition (7 traces with 110 frames each) and baseline. Black vertical lines indicate thresholds of the *p* < 0.05 criterion for activity differing significantly from baseline. **(D)** Histogram of filtered differences between P cell responses to stimulated condition (7 traces with 110 frames each) and baseline. Black vertical lines indicate the significance thresholds determined in **(C)**, showing that the activity differs significantly from baseline more often than in control condition. The activity map in **(B)** depicts in which frames the significant deviations from baseline occurred, indicating consistent activation during current stimulation.

Voltage sensitive dye (VSD) recording was performed in isolated leech midbody ganglia simultaneously to a double intracellular recording from a P and a T cell. Both mechanosensory cells were stimulated with intracellular current injection, while the activities of all visible cells on the ventral side of the ganglion were monitored through a microscope [Zeiss Examiner.D1, objective plan-apochromat 20 x/1.0 DIC (UV)] with a CCD camera (Photometrics QuantEM:512SC), using bath-applied VF2.1.CL dye (λ_max_ = 522 nm, λ_em_ = 535 nm, see Miller et al., 2012). Imaging was performed with a temporal sampling frequency of 94 Hz and a spatial resolution of 64 × 128 pixels. Prior to the recording, a snap shot was taken with the full spatial resolution of the camera, 512 × 512 pixels (Figure 2A), based on which regions of interest (ROIs) representing individual cell bodies were selected manually (Fathiazar et al., 2016; see **Figure 7B** for an example). In this manuscript, data from one representative VSD recording is presented. Similar results were obtained in seven additional preparations.

Electrical stimulation, consisting of 10 ms long pulses of 2 nA (T cell) or 3 nA (P cell), was designed to mimic these cells' spiking patterns in response to tactile stimulation of 70 mN in the ventral midline of the skin in a semi-intact preparation (Pirschel and Kretzberg, 2016). Four different stimulus conditions were compared (see **Figure 7**): In the PT-stimulated condition, both sensory cells were electrically stimulated in a pattern that reproduces natural responses to tactile stimulation. In the P-stimulated and the T-stimulated condition only one of the cells was stimulated, while the other cell remained unstimulated. In control condition, both cells were not stimulated. In our experiments, responses to 7 repetitions of each condition (trials) were recorded.

For data analysis, 55 ROIs corresponding to visible cell bodies were drawn over the first frame of the VSD recording presented in this manuscript. VSD signals of the cells were extracted by averaging and normalizing the brightness of the pixels in the corresponding ROIs. Movement and bleaching artifacts were corrected as described in Fathiazar and Kretzberg (2015). For each cell, baseline (black line in Figure 2B) was calculated as an average of the seven trials of control condition. Baseline was subtracted from all VSD signals obtained for all four stimulus conditions. To reduce the noise level, the difference signal was filtered with a moving average filter of three frames window size.

Statistical analysis to identify stimulus-activated cells was performed as described in Fathiazar et al. (2016). In short, the histogram of the filtered VSD difference signals in control conditions was calculated for each cell. Applying a statistical significance level of α = 0.05 on this histogram, we defined the thresholds of activity differing significantly from baseline (black vertical lines in Figure 2C), indicating very strong de- or hyperpolarization of the cell's membrane potential. These thresholds (quantiles 2.5 and 97.5% of control response distribution) were applied to the filtered VSD difference signals obtained for the three conditions of mechanoreceptor stimulation (Figure 2D: P-stimulated condition) to discriminate which individual cells were activated at each time frame (activation map of the P cell in Figure 2B). The activation maps I(i,j) in **Figures 7C–F** show the pooled activity for all seven trials of each condition, where I(i,j)ϵ{0, …,7} and iϵ{1, …,55} indicates the cell number (chosen by the sequence of cells' activations after stimulus onset, not corresponding to the cell numbers in the standard ganglion map shown e.g., in Lockery and Kristan, 1990b) and jϵ{1, …,110} is the frame (referring to times 0.16 < *t* < 1.36 in s). I(i,j) has the value of 0 (shown in dark blue) if the cell i in frame j was not activated in any of the seven trials. If cell i was found to be activated in all the trials in frame j, I(i,j) has the value of seven (shown in yellow). A cell i was classified as a “stimulus-activated” cell for a specific stimulus condition (PT-, P-, or T-stimulated), if at least six of the seven trials revealed significantly increased or decreased activity compared to baseline in at least one time frame in the period 0.53 < *t* < 0.87 s (from stimulus onset to offset plus five frames).

## Results

### Encoding of tactile information by mechanoreceptors

**Figure 3:**
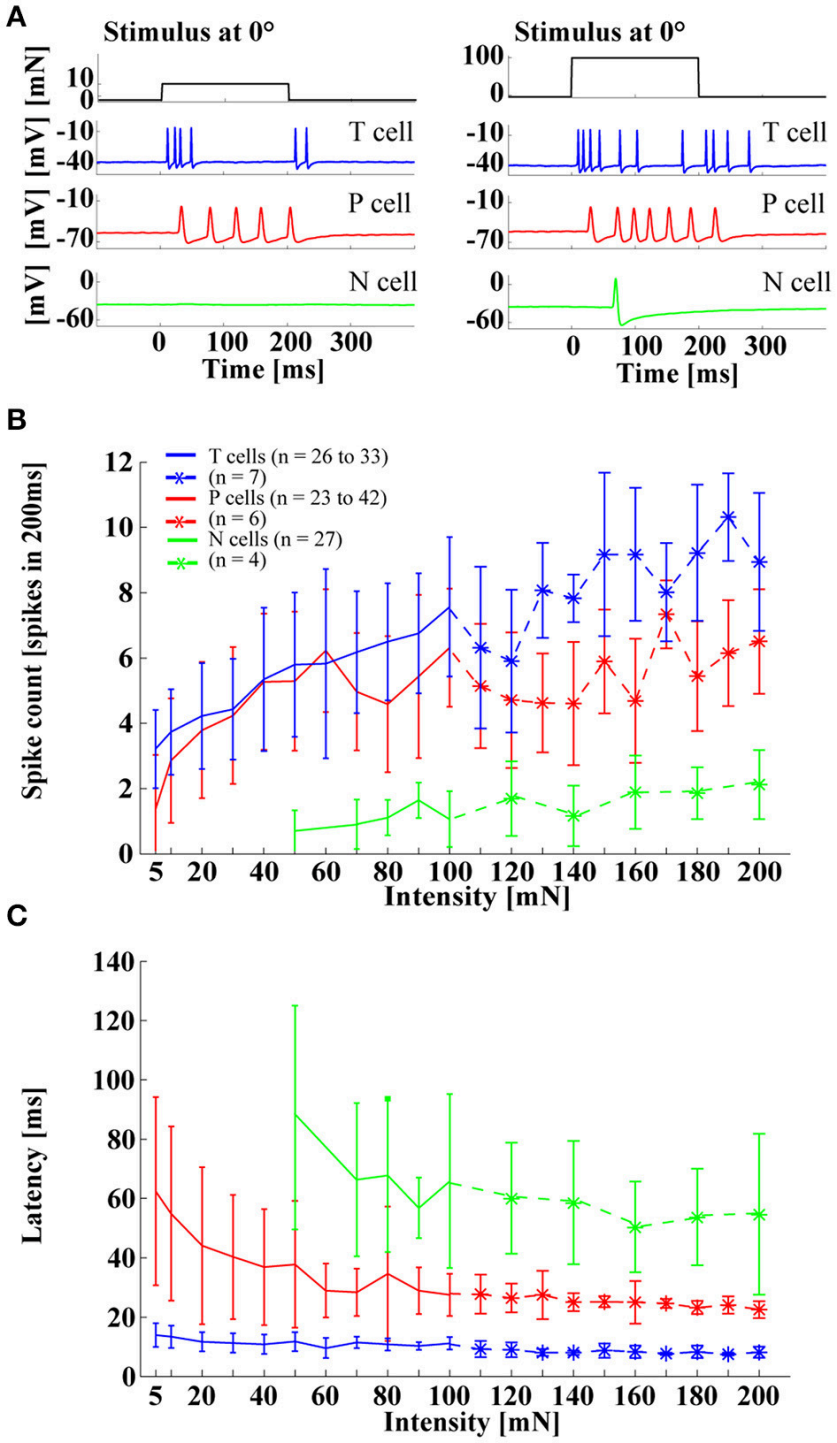
**Influences of stimulus intensity on mechanoreceptor responses. (A)** Example responses of an intracellular triple recording of a left T_v_ cell (blue), a left P_v_ cell (red), and a right N cell (green) responding to a tactile stimulus of 10 mN (left) and 100 mN (right) applied for 200 ms at 0° (ventral midline). **(B)** Spike count and **(C)** response latency (mean and STD) for T_v_ cells (blue), P_v_ cells (red) and N cells (green) responding to tactile stimuli with intensities of 5–200 mN applied at 0°. Stronger pressure intensities (> 100 mN) were tested with fewer cells (see legend).

The three types of leech mechanoreceptors were classically associated with tactile stimuli of different intensities, as reflected in their notation: T cells for light touch, P cells for stronger pressure, and N cells for noxious, very hard mechanical stimulation (Nicholls and Baylor, 1968). However, simultaneous recordings of different mechanoreceptor types responding to skin stimulation revealed a different picture: Both T and P cells responded reliably to a large range of stimulus intensities, from very light touch (5 mN) to strong pressure (200 mN), and even N cell responses started at a moderate touch intensity of 50 mN (Figure 3). These strongly overlapping sensitivity ranges clearly contradicted the classical idea of a labeled line code with different cell types, signaling the presence of stimuli in distinct force intensity ranges. Instead, this finding suggested that the tiny population of leech mechanoreceptors (6 T cells, 4 P cells, 4 N cells in each ganglion) uses a different strategy for encoding the intensity of tactile stimuli. As shown in Figure 3A, response patterns to tactile stimulation at the ventral midline differed considerably between cell types, in accordance with many previous publications (Nicholls and Baylor, 1968; Carlton and McVean, 1995; Lewis and Kristan, 1998b; Pirschel and Kretzberg, 2016). T_v_ cells typically produced transient, rapidly adapting responses, both at stimulus onset and offset, while P_v_ cells usually responded with sustained sequences of regularly occurring spikes within the entire duration of tactile stimulation. N cells were not very active when the skin was stimulated with relatively weak pressure, leading to responses consisting of only one or two spikes. Despite these differences in spike timing patterns, all three types of mechanoreceptors shared similar dependencies of standard response features on stimulus intensity. All cells responded to increasing pressure intensity with increasing spikes counts and decreasing response latencies, both of which saturated for high intensities (100–200 mN) in T and P cell responses. In a preceding study (Pirschel and Kretzberg, 2016), we showed for the intensity range of 5–100 mN that summed spike counts of mechanoreceptor pairs yielded the best estimation performance for stimulus intensity. In particular the sustained P_v_ cell responses allowed a reliable estimation.

**Figure 4:**
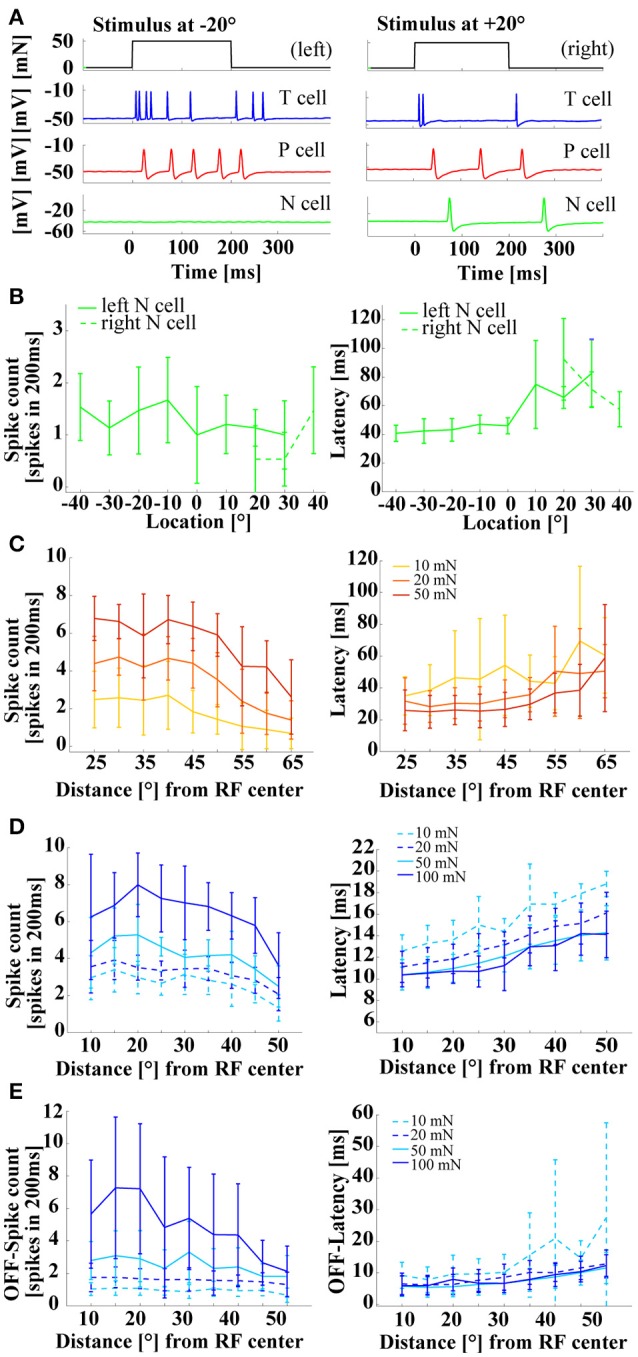
**Influences of stimulus location on mechanoreceptor responses. (A)** Example responses of an intracellular triple recording of a left T_v_ cell (blue), a left P_v_ cell (red), and a right N cell (green) responding to a tactile stimulus of 50 mN for 200 ms at locations −20° (left) and +20° (right). Ventral midline is defined as 0°, stimulus locations to the right as positive and to the left as negative numbers of degrees. **(B)** Example for the dependency of N cell spike count and response latency on stimulus location. Spike count and response latency (mean and STD) are shown for one representative double recording of two N cells with 15 repeated stimulus presentations and a stimulus intensity of 100 mN. **(C)** Dependency of spike count and response latency (mean and STD) of P_v_ cells (*N* = 10, each 8–10 stimulus presentations; pooled responses of left and right cells) on stimulus location. Responses at different locations [displayed as distance from receptive field center in (°)] are shown for three stimulus intensities of 10 mN (yellow), 20 mN (orange), and 50 mN (red). **(D)** Dependency of spike count and latency (Mean and STD) of T_v_ cells (*N* = 10, each 8–10 repeated stimulus presentations; pooled responses of left and right cells) on stimulus location. Responses at different locations [displayed as distance from receptive field center in (°)] are shown for four stimulus intensities of 10 mN (dashed-cyan), 20 mN (dashed blue), 50 mN (solid-cyan), and 100 mN (solid-blue) (*N* = 8 cells). **(E)** Dependency of off-spike count and off-spike latency (Mean and STD) of T_v_ cells on stimulus location (same recordings and figure conventions as in **D**). Linear fits for the stimulus response curves shown in **(D**,**E)** are provided in the Supplementary Material.

When stimulus location was varied, P_v_ and T_v_ cells showed the same effects as were reported in previous studies (Thomson and Kristan, 2006; Pirschel and Kretzberg, 2016). Spike rates decreased and latencies increased with increasing distance from the center of the cell's receptive field (Figures 4A,C,D). A similar tendency was also visible for N cell responses (Figure 4B), although the low spike counts (between 0 and 2 spikes in 200 ms), induced by the range of stimulus intensities applied in this study, made results more difficult to interpret. In Pirschel and Kretzberg (2016) it was shown that for a tactile stimulation with 50 mN, the latency differences between pairs of mechanoreceptors, in particular the fast responses of T_v_ cells, led to the best location-estimation performance. Here, we extended the analysis of P_v_ and T_v_ cell responses by varying combinations of stimulus location and force intensity, while keeping velocity and all other stimulus parameters constant across experiments. Stimuli of all intensities yielded similar dependencies of spike counts and latencies on stimulus location, with higher mechanical force triggering more and earlier spikes, resulting in virtually parallel curves for both response features (Figures 4C,D). For T_v_ cells similar response characteristics of spike counts and latencies were found even for strong pressure stimuli of 100 mN (Figure 4D), giving further evidence against a labeled line coding of stimulus intensities.

**Figure 5:**
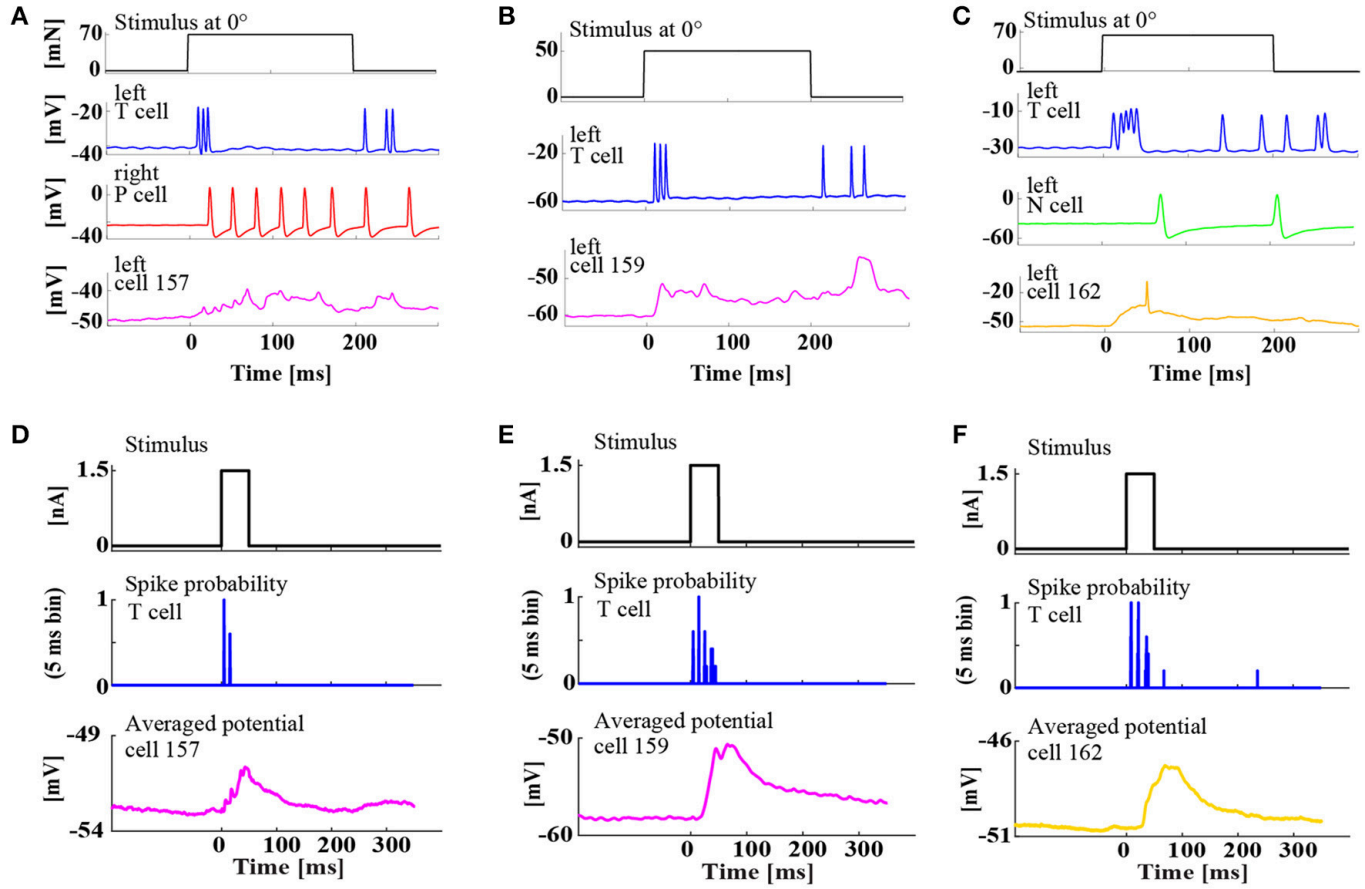
**Simultaneous, intracellular recordings of three different interneurons and ipsilateral mechanoreceptors. (A–C)** Example responses of mechanoreceptors and three types of interneurons to tactile skin stimulation for 200 ms at 0° (ventral midline). **(A)** Triple recording of left T_v_ cell (blue), right P_v_ cell (red), and left cell 157 (magenta) responding to a tactile stimulus of 70 mN. **(B)** Double recording of left T_v_ cell (blue) and left cell 159 (magenta) responding to a tactile stimulus of 50 mN. **(C)** Triple recording of left T_v_ cell (blue), a left N cell (green), and left cell 162 (yellow) responding to a tactile stimulus of 70 mN. **(D–F)** Responses of the same interneurons of types **(D)** cell 157, **(E)** cell 159, **(F)** cell 162 to constant current injection of 1.5 nA for 50 ms (upper trace) applied intracellularly to the ipsilateral T_v_ cell, which responded reproducibly with regular spike patterns (see spike probability in 5 ms bins) in the same double or triple recording as shown in the corresponding upper panel **(A–C)**. Cell responses were averaged over 20–50 repetitions.

Since T_v_ cells also responded to the offset of a constant tactile stimulation (Figures 3A, 4A, 5A–C), stimulus force intensity and location dependencies of these off-responses were also analyzed (Figure 4E). Only strong pressure stimuli (100 mN) close to the receptive field center triggered large numbers of off spikes in T_v_ cells. These off response spike counts decreased steeply with distance (Figure 4E left, Supplementary Figure 1C). For light and moderate tactile stimulation, off-response spike counts were lower then spike counts at stimulus onset. These off-response spike counts depended mainly on stimulus intensity, while stimulus location had virtually no effect, resulting in the parallel flat curves shown in the left panel of Figure 4E. Consequently, linear regression revealed shallower decreases and smaller y-intercepts of spike counts at stimulus offset (Supplementary Figure 1C) than at stimulus onset (Supplementary Figure 1A, see also Table 1 in Supplementary Material for comparison). In contrast, the latency of off-responses triggered by moderate and high mechanical force depended almost exclusively on stimulus location (Figure 4E right panel, Supplementary Figure 1D). The virtually identical off latency response curves obtained for intensities between 20 and 100 mN rose at least as steeply with increasing distance from the center of the receptive field as for the latencies observed at stimulus onset (Supplementary Figures 1B,D, Supplementary Table 1) and showed similarly low variability (Figures 4D,E right panels). Only very soft touch stimuli of 10 mN, which often failed to trigger off-responses at all, caused highly variable off response latencies, which were not approximated well by linear regression (Supplementary Figure 1D). In conclusion, these results suggest that T cell responses occurring at the offset of skin stimulation could play an additional role for tactile encoding.

### Interneurons involved in tactile information processing

After studying the encoding of tactile stimulus properties at the mechanoreceptor level, the main questions arising from these results are: Which mechanoreceptors provide input to which of the cells at the next network level? And which mechanoreceptor response features shape the responses of which interneurons involved in processing tactile information?

To tackle these questions, we performed a combination of three experimental approaches: Simultaneous intracellular double recordings from a mechanoreceptor and an interneuron, anatomical examination revealing potential contact points, and voltage sensitive dye recordings providing access to mechanoreceptor-induced responses of many cells simultaneously.

In the first step, responses of three different interneurons were characterized by the classical electrophysiological approach: Intracellular double and triple recordings of mechanoreceptor(s) and an interneuron (Figure 5). The three interneurons 157, 159, and 162 (see Figure 1B for cell body positions in the ganglion) were previously identified as members of the local bend network according to the criteria that they responded to presynaptic P cell stimulation and influenced the activity of motor neuron 3 (Lockery and Kristan, 1990b). Here, our recordings showed that these three interneuron types also receive synaptic input from an ipsilateral T_v_ cell. Intracellular injection of a constant current step reproducibly triggered rhythmic spike patterns in T_v_ cells, which elicited clear excitatory postsynaptic potentials (EPSPs) in all three types of interneurons (Figures 5D–F).

Additionally, intracellular recordings of interneurons 157, 159, and 162 during tactile stimulation provided direct evidence that these cell types are involved in the processing of tactile information (Figures 5A–C). The recordings revealed their distinctly different response characteristics: Cell 157 displayed a sustained graded response, resembling integrated EPSPs lasting for the entire duration of stimulation (Figure 5A), while the other two cell types responded more transiently. Cell 159 produced large EPSPs both at tactile stimulus on- and offset (Figure 5B). Cell 162 responded mainly with a very large EPSP at stimulus onset, sometimes triggering a single postsynaptic spike (Figure 5C).

**Figure 6:**
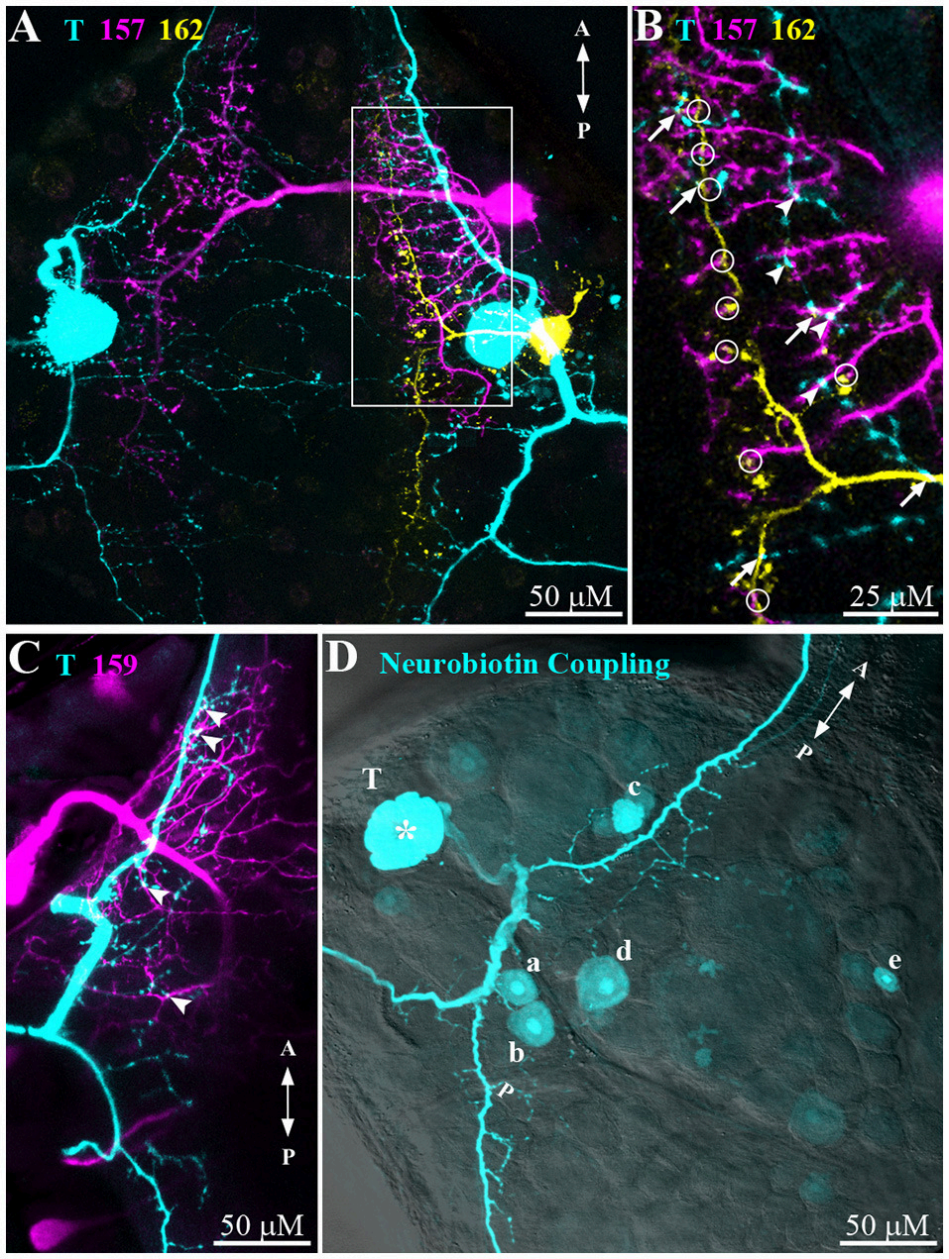
**Morphological connections of T cells and interneurons. (A)** Dye injections (Alexa Fluor 488/546/633) were performed to reveal the morphology and putative cell-cell contact zones of a T cell (cyan), interneuron 157 (magenta), and 162 (yellow). Z-depth 70 μm. **(B)** Magnification of the area indicated in **(A)** (white box) shows putative contacts between T cell and interneuron 157 (arrowheads), T cell and interneuron 162 (arrows), and interneuron 157 and 162 (circles). Z-depth 10 μm. See Supplementary Material for an animation of the confocal image stack underlying this figure. **(C)** Visualized morphology of an Alexa-dye injected T cell (cyan) and a Neurobiotin injected interneuron 159 (magenta). Arrowheads indicate putative contacts. Z-depth 30 μm. **(D)** Neurobiotin (cyan) was injected into a T cell (indicated by asterisk) to reveal electrically coupled cells. Putative cell types: (a) and (b) interneurons 62 and 61, (c) 159, (d) 212, and (e) unknown. Z-depth 110 μm. Confocal microscope transmission image overlayed with 40% transparency for cell location identification. In all panels letters A and P indicate anterior to posterior direction of the ganglion.

The second step of the network analyses provided anatomical evidence for network connections (Figure 6). Simultaneous dye injections into T cells and interneurons revealed cell morphology and prospective points of contacts. Potential locations of contacts with a T cell (cyan) were found for all three types of interneurons 157 (magenta, arrowheads in Figure 6B), 159 (magenta, arrowheads in Figure 6C), and 162 (yellow, arrows in Figure 6B). Interestingly, the triple staining of T, 157, and 162 (see also stack animation in Supplementary Material) additionally identified putative contacts of interneurons 157 and 162 suggesting potential lateral network connections at the interneuron level (circles in Figure 6B). However, since the study by Lockery and Kristan (1990b) did not find synaptic responses in double recordings of this cell pair, additional electrophysiological tests are needed.

Neurobiotin injection into a T cell (Figure 6D) led to staining of five additional cell bodies suggesting electrical coupling. For one of them, the location of the cell body matched the location of cell 159 (labeled c in Figure 6D), fitted very well with the electrophysiological finding of this cell type's responses following the time course of T cell responses (Figures 5B,E). Judging from the cell body location one of the other cells could be cell 212 (labeled d in Figure 6D), which was also identified as local bend interneuron by Lockery and Kristan (1990b). Two more cells were stained in the posterior-lateral package of the ganglion. By location these cells could be numbers 61 and 62 (b and a in Figure 6D) in the standard ganglion map (Figure 1B). At a larger distance from the T cell an additional cell body (e in Figure 6D) was also clearly stained, but remained to be identified.

**Figure 7:**
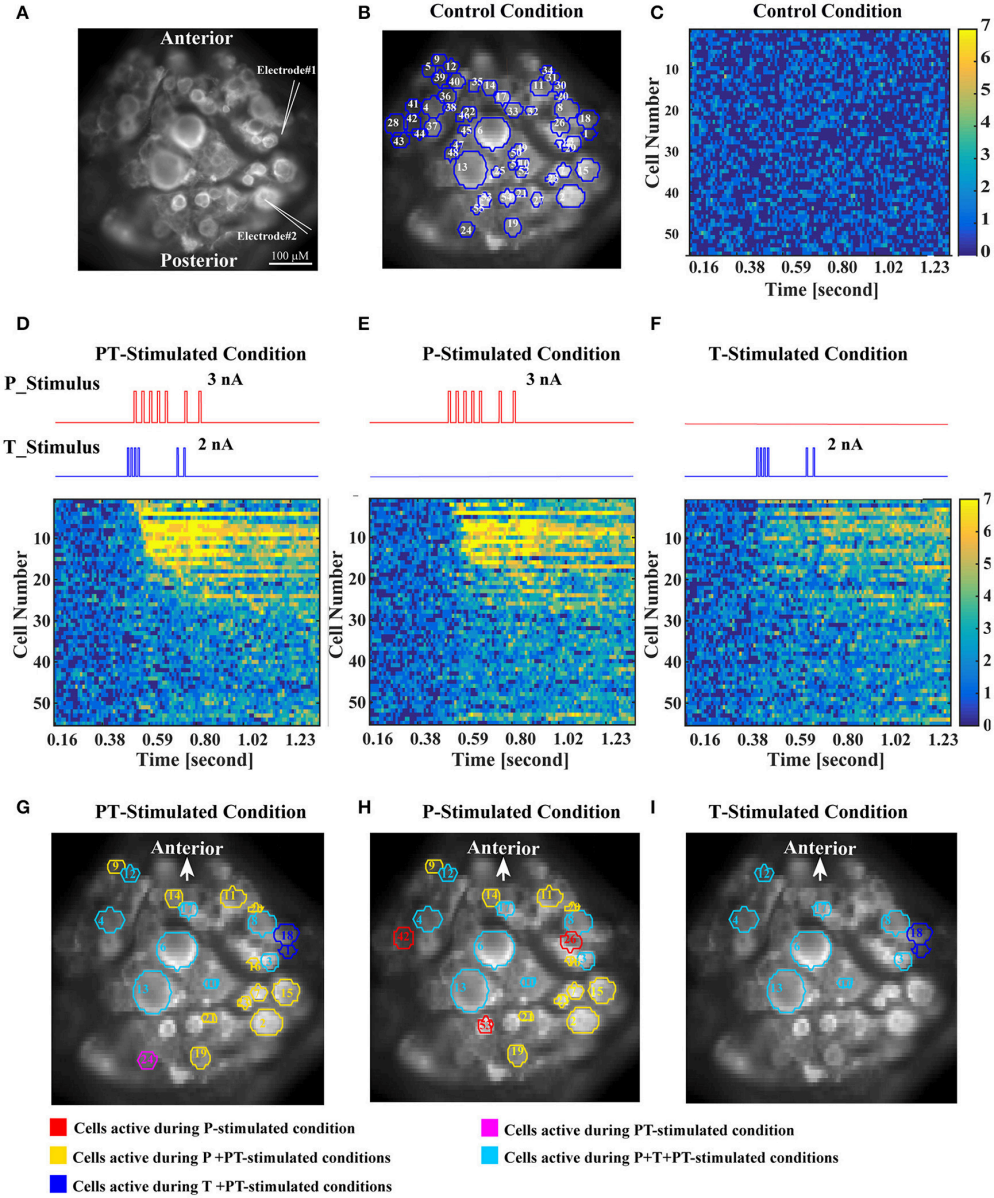
**Identification of interneurons involved in the processing of tactile stimuli based on VSD recordings. (A)** High resolution image of VSD labeled cells in the leech ganglion. For typical positions of the cell bodies located on the ventral surface of the ganglion, refer to Figure 1B. Intracellular electrodes (symbolized by white pointed angles) were used for electrical stimulation of a T cell (Electrode #1) and a P cell (Electrode #2). **(B)** One frame of the VSD recorded ganglion with superimposed blue cell borders showing all 55 ROIs used for analysis. White numbers refer to the order of cells' activation determined in **(D)**, not to the cell identity numbers commonly used in the standard ganglion map. **(C)** Activity map of all 55 recorded cells in response to control condition (no stimulation). The color of each pixel indicates in how many of the seven control trials the activity of a specific cell (row) at a specific recording frame (column) deviated significantly from baseline. Colors range from dark blue (0 deviations) to yellow (7 deviations). The absence of bright colors indicates that no consistent deviation from baseline occurred for any of the cells. Cell numbers correspond to **(B,D)**. **(D)** Activity map in response to intracellular current stimulation of a P cell and a T cell (stimulus time courses shown above in red and blue). Cells were sorted and numbered by the timing of the first occurrence of consistent significant deviation (>5 of 7 trials) from baseline in this condition after stimulus onset (T cell is #1, P cell is #2). Cells not activated by the PT-stimulated condition remained in random order. For cell body locations see **(B)**. **(E,F)** Activity maps in response to intracellular current stimulation of only the P cell **(E)** or the T cell **(F)**. Cell numbers correspond to **(B,D)**. **(G–I)** Cells activated by the specific stimulus conditions, P and T cell stimulation **(G)**, only P cell stimulation **(H)**, and only T cell stimulation **(I)**. A cell was defined as stimulus activated, if its activity deviated significantly from baseline in more than 5 of 7 trials, in at least one time frame after stimulus onset (see Methods). Red ROIs show cells activated only during P stimulated condition, yellow ROIs during P and PT conditions, blue during T and PT conditions, magenta only during PT condition, cyan during all three conditions.

The third step of our analyses aimed to identify interneurons involved in tactile processing using voltage sensitive dye (VSD) recordings. In these experiments, intracellular double recordings of a T and a P cell were performed, while the activity of the ventral side of the ganglion was imaged. After VSD bath application, graded de- and hyperpolarization of all neurons could be estimated based on the emitted light of the corresponding pixels in the camera image (Miller et al., 2012). Individual spikes could only be identified in VSD traces of some cell types with large and slow spikes, otherwise the temporal resolution of the camera (94 Hz at a spatial resolution of 64 × 128 pixels) and the signal to noise ratio were too low. In the recording shown in Figure 7, 55 ROIs representing individual cell bodies were selected for analysis (Figure 7B). Four different conditions of electrical stimulation were used during VSD recordings: (1) Control condition without stimulation (Figure 7C), used to determine baseline spontaneous network activity, (2) PT-stimulated condition (Figures 7D,G) with short current pulses injected into the cell bodies of the T cell and the P cell, which elicited spike trains reproducing typical mechanoreceptor responses to a touch stimulation (Pirschel and Kretzberg, 2016), (3) P-stimulated condition (Figures 7E,H) with the same spike train elicited in the P cell as in the PT-stimulated condition, while the T cell remained unstimulated, and the corresponding (4) T-stimulated condition (Figures 7F,I) with only T cell stimulation.

For the statistical analysis to identify interneurons activated by mechanoreceptor responses the control condition was used to calculate the normal range of spontaneous activity for each cell. The upper and the lower thresholds of this range were defined as percentiles 2.5 and 97.5% of the empirically determined distribution of VSD values (leading to a significance level of α = 0.05, see Section Methods, Figure 2C, and Fathiazar et al., 2016) These thresholds were applied to the same cell's responses during the three different stimulated conditions to find if and when the cell was more de- or hyperpolarized than during control condition (see Figure 2). Figures 7D–F show for each cell at each time frame how many of the seven trials deviated significantly from baseline, with a color code ranging from dark blue (no deviations from baseline) to yellow (deviation from baseline in this time frame in all seven stimulus presentations). In Figure 7 cells were numbered according to the timing of their first activation (significant deviation from baseline in at least six of seven stimulus presentations) after stimulus onset in the PT-stimulated condition (Figure 7D). Hence, cell numbers in Figure 7 differ from the cell identity numbers used in the standard ganglion map, e.g., in Figure 1B and in Lockery and Kristan (1990b). In Figure 7 cell number 1 is the stimulated T cell, cell number 2 the stimulated P cell. Cells showing consistent deviations from the baseline during stimulation in at least six out of seven presentations were classified as stimulus-activated (see Section voltage sensitive dye experiments and analysis). These cells are indicated by colored borders in Figures 7G–I.

Comparison of different stimulation conditions revealed that electrical stimulation of T and P cell together (Figure 7D) as well as activation of P cell alone (Figure 7E) activated 22 of the 55 analyzed cells, while T cell stimulation alone elicited significant activation only in 10 cells. However, populations of activated cells were not identical for PT-stimulated and for P-stimulated conditions. One cell (number 24, magenta in Figure 7G) reached activation threshold exclusively when it received input from both the P and the T cell. Since it was located in the posterior package and was not activated by P cell input alone, this cell did not correspond to any of the known local bend interneurons (Lockery and Kristan, 1990b). In addition to the T cell (number 1) itself one cell (number 18, blue in Figures 7G,I), putatively interneuron 161, needed T cell but no P cell stimulation for activation. Interestingly, three cells [numbers 26, 32, 42 (Figure 7H red), cell types remained to be identified] showed significant activity during P cell stimulation, but not in response to the combined PT-stimulated condition. This finding could indicate nonlinear interaction of inputs from different mechanoreceptors or inhibition by the T cell.

According to our classification criterion, eight cells responded with consistent significant activation to all three stimulated conditions (cyan borders in Figures 7G–I). Some of them were easy to identify by soma positions and sizes and by their characteristic response patterns: Both Retzius cells (numbers 6 and 13 in Figure 7) and both AP cells (numbers 4 and 8 in Figure 7) could be identified unambiguously across different preparations. The fastest postsynaptic responding cell, marked with number 3, could be the ipsilateral interneuron 162 (compare Figures 5, 6). The cell labeled with number 10 could putatively be interneuron 212 (compare Figure 6D and the ganglion map in Figure 1B). Cells labeled 12 and 17, which also were activated in all three stimulated conditions, still remained to be identified.

Some of the interneurons identified by the study of Lockery and Kristan (1990b) could correspond to the cells activated by PT- and P-stimulated conditions (cyan borders in Figures 7G,H). In particular, judging by position, the cells labeled with numbers 11 and 20 could correspond to the interneurons 157 and 159 analyzed in this study. These cells were not found in the map of the activated cells in the T-stimulated condition shown in Figure 7I even though the connection to a T cell was demonstrated both electrophysiologically (Figure 5) as well as anatomically (Figure 6). However, this discrepancy seemed to be due to the very strict criterion for the classification of T-condition activated cells requiring precisely timed and strong deviation from baseline activity in at least six of the seven trials. Since interneuron response amplitudes were small and VSD signal-to-noise ratio was low this criterion provided a conservative estimate of cells showing very clear responses. When this criterion was relaxed by a lower significance level or a lower number of significant trials used as threshold, more cells, including the interneurons under study, were classified as activated (results not shown). In future studies, network activation patterns obtained for varied classification criteria need to be compared across different preparations to reveal all members in the network and the interaction of different types of mechanosensory inputs.

## Discussion

After more than 30 years of research on the local bend reflex of the leech (Kristan, 1982), the perspective on the neuronal network controlling this seemingly simple behavior still gains complexity. In line with other recent findings (Gaudry and Kristan, 2009; Palmer et al., 2014; Baljon and Wagenaar, 2015; Pirschel and Kretzberg, 2016) the results of this study indicate that the model of the local bend network needs to be revised regarding the input signals provided by mechanoreceptors and the computation performed by interneurons.

### Encoding of tactile information by mechanoreceptors

On the level of mechanoreceptors our results suggest that three dogmata of leech tactile information processing should be revised:

Contrary to the common belief (and the cell's names), the three types of mechanoreceptors—touch, pressure and noxious cells—do not implement a labeled line code for tactile stimulus intensities. The ranges of constant pressure intensities encoded by these cell types overlapped quite substantially, with longer stimulus durations leading to higher spike counts (Pirschel and Kretzberg, 2016). T and P cells both reacted to the entire range of tested intensities from very light touch (5 mN) to moderate pressure (200 mN) with increasing spike counts and decreasing response latencies. In both cell types N cells generated spikes in response to moderate stimulus intensities. Even though the relatively weak tactile stimuli used in this study were clearly not in the optimal range for N cell stimulation, they elicited weak but reliable N cell responses. Indeed, a large range of intensities triggered spikes in all three cell types and also N cells might contribute to the local bend network by providing additional input to interneurons.

Despite the finding that T cell spikes increase muscle tension during the local bend response reported already in the first publication on leech local bending (Kristan, 1982) their contribution to the network was disregarded in most studies. Electrical stimulation of a single P cell was sufficient to elicit a local bend response, while a single T cell often failed to trigger an obvious muscle movement. It was therefore concluded that the local bend network relies on P cell rather than T cell input (Kristan, 1982; Lewis and Kristan, 1998b). However, recent results suggest that T cells encode tactile stimulus properties by relative response features of a cell pair with overlapping receptive fields (Pirschel and Kretzberg, 2016). Thus, electrical stimulation of a single T cell triggers a response that would not occur in natural situations. Each patch of skin is innervated by a pair of T cells and a pair of P cells with overlapping receptive fields. They all respond to tactile stimulation at this location (see Figure 1A bottom for a sketch of overlapping receptive field at ventral midline). Hence, even if spikes of a single stimulated cell fail to elicit the local bend response it cannot be concluded that this cell is not important for the response. Carlton and McVean (1995) pointed out that T cells provide behaviourally important input to the leech nervous system, in particular when acting as velocity detectors in exploration behavior. In a study comparing T and P cell encoding (Pirschel and Kretzberg, 2016), T cell responses were shown to allow higher percentages of tactile stimulus location estimation than P cell responses. Moreover, mixed populations of P and T cells considerably improved the combined estimation of stimulus location and intensity compared to each cell type separately. Here, we showed with three complementary methods that T cells provide synaptic input to several previously identified local bend interneurons (Figures 5–7). Hence, T cells should be considered as additional members of the local bend network.

As in most neuronal systems, the analysis of leech mechanoreceptor responses was restricted to spike counts of single cells for many decades. However, more recent studies showed that combining responses of two cells with overlapping receptive fields drastically improves stimulus estimation and that temporal response features contain more information about stimulus location than spike counts (Thomson and Kristan, 2006; Pirschel and Kretzberg, 2016). This study confirmed that spike counts and response latencies depended both on stimulus intensity and location for T_v_ and P_v_ cells and showed similar dependencies also for N cell responses (Figures 3, 4). Moreover, combined variation of stimulus location and intensities revealed that the dependency of both response features on stimulus location stayed the same for different stimulus intensities, leading to parallel shifted tuning curves in Figures 4C,D. Since T_v_ cells produced transient responses at stimulus on- and offset, encoding properties of off-responses occurring after stimulus offset were additionally analyzed. Interestingly, the off-response spike count showed a much stronger dependency on stimulus force intensity than on location—at least for light and moderate tactile stimulation. In contrast, off-response latency depended almost exclusively on stimulus location. This finding suggests that T cell off-spikes could play an additional role in tactile information encoding that should be considered in future studies. For primate afferents, on-off-response patterns were proposed to play a role in the encoding of object contact and release during active touching (Johnson, 2001; Johansson and Flanagan, 2009). Hence, the importance of T cells during exploration (Carlton and McVean, 1995)—actively touching the environment—might indicate a general mechanism of tactile stimulus encoding shared by man and worm.

Taken together, these results suggest that encoding of tactile stimulation on the mechanoreceptor level can be explained neither by a labeled line of different cell types encoding distinct ranges of mechanical force, nor by symmetrical spike count tuning curves representing stimulus location. Instead, we propose a mixed-type population of mechanoreceptors performing simultaneous encoding of stimulus location and intensity by multiplexing temporal response features and spike counts. Since mixed-type combination of multiple afferent classes and multiplexed encoding of several stimulus properties were also proposed as underlying mechanisms of touch perception in primates (Saal and Bensmaia, 2014), these encoding principles might be fundamental mechanisms of tactile information processing. For the leech, future studies are needed to investigate how additional stimulus properties like probe shape and velocity are represented in this mixed-type, multiplexed coding scheme.

### Interneurons involved in tactile information processing

Any sensory system relies on receptors conveying all available information about the stimulus to the next network level. In many systems, including the mechanoreceptors of primates (Saal and Bensmaia, 2014) and leeches (Nicholls and Baylor, 1968; Carlton and McVean, 1995), this input layer of the sensory processing network contains different receptor types (Smith and Lewin, 2009), which specifically react to certain types of stimulation. However, for exploiting the information about the stimulus encoded by receptors, this information must be transferred to and processed by the next network layers. While it is difficult to study directly connected pre- and postsynaptic cells in complex sensory systems in vertebrates like the primate, the individually characterized cells in the simple nervous system of the leech are optimally suited for this question.

As discussed in section Encoding of Tactile Information by Mechanoreceptors, our hypothesis is that the individual sensory cells send multiplexed signals, containing a combination of temporal response features and spike rate, which simultaneously represent multiple stimulus properties. The ensemble of interneurons has the task to integrate and process these ambiguous signals coming from the 10 mechanoreceptors (6 T, 4 P, 2 N), which are present in each ganglion. Our preliminary results suggest that the individual interneurons have spatial receptive fields as was also found by Lockery and Kristan (1990b), but additionally differ in their integration properties. At least one type of interneuron (cell 157, Figure 5A) seemed to act as slow integrator, presumably reacting mainly to the spike count of all presynaptic cells. The membrane potentials of other interneurons (cells 159 and 162, Figures 5B,C) showed more complex temporal response structures, suggesting temporal information processing, e.g., as coincidence detectors. Furthermore, these results indicate that responses of individual interneurons could be influenced to different extents by responses of the three mechanosensory cell types. While the responses of slow integrators probably follow mainly the sustained P cell spikes, the more complex interneuron response patterns could stem from the transient T cell responses to stimulus changes and the sparse N cell spikes.

Interneuron responses found in this study matched and complemented previous findings. The three interneurons considered here in more detail, cells 157, 159, 162, were identified as local bend interneurons, receiving P cell input and influencing motor neuron activity (Lockery and Kristan, 1990b). Judging by locations of cells' somata, all of these three interneurons also significantly changed their membrane potentials when a P cell was stimulated in our voltage sensitive dye recordings (Figures 7G,H). Furthermore, our physiological and anatomical results (Figures 5, 6) showed that these three cells also receive input from T cells.

In addition to these three interneurons, which we chose to study in detail, our results showed several other cells receiving mechanoreceptor inputs, confirming results from previous studies. Judging by location of their cell bodies, our VSD experiments yielded at least two more previously identified local bend interneurons, cells 161 and 212 (Lockery and Kristan, 1990b), reacting to T-cell stimulation (Figure 7I). Cell 212 might also be one of the cells visible in the Neurobiotin staining of a T cell, indicating electrical coupling (Figure 6D). Another interneuron, cell 61, for which we found a putative electrical coupling to the Neurobiotin-filled T cell and an activation in the VSD experiments, also was reported before to receive mechanoreceptor input (Nusbaum and Kristan, 1986). Activity of this serotonin-containing cell was associated with modification of the local bend behavior and initiation of swimming (Nusbaum and Kristan, 1986; Kristan et al., 1988; Lockery and Kristan, 1991). Moreover, the activation of Retzius and AP cell pairs in our VSD experiments (Figures 7G–I) was also consistent with previous findings that both cell types react to mechanoreceptor responses and pressure applied to the skin (Zhang et al., 1990; Lockery and Kristan, 1991; Zhang et al., 1995; Jin and Zhang, 2002; Fathiazar et al., 2016).

Despite this updated list of candidate cells revealed in this study, we assume that not all interneurons involved in processing of tactile information showed up as stimulus activated cells in the VSD experiments (Figures 7G–I), because of three technical reasons: (1) the restricted visibility of cells in preparations, (2) the statistical selection criterion, and (3) the type of stimulation used in this study.

Firstly, visibility of cells in VSD recordings varies from preparation to preparation. VSD experiments require removal of the glia sheath from the ganglion to ensure that the dye reaches all neuronal membranes. However, this procedure led to displacement of the cell bodies. Some cell bodies moved out of focus of the microscope. Proximate cells, which are usually well visible in the ganglion before de-sheathing, might overlap or even completely occlude each other after that dissection procedure. These effects led to a lower number of ROIs (55 in Figure 7B) visible in the VSD images than cells located at the ventral side of the ganglion (approximately 200). Moreover, even though the positions of cell bodies in the ganglion are relatively fixed, they sometimes switch positions, requiring additional physiological or anatomical evidence for definite cell type classification. Hence, it is of general concern that not all stimulus-activated interneurons can be found in all VSD preparations.

The second reason for the low number of interneurons classified as stimulus-activated (in particular for separate T-cell stimulation, Figures 7F,I) is the strict criterion we applied. A cell's activity needed to deviate significantly (α = 0.05) from baseline activity in at least in six out of seven stimulated trials in exactly the same frame. Hence, in this time frame the cell had to be consistently more depolarized or more hyperpolarized than 97.5% of the values obtained under control conditions. Relaxing this criterion led to a higher number of cells classified as stimulus-activated. Example, for a level of α = 0.1 and the same threshold (six out of seven active trials), 40 cells were marked as stimulus-activated in the PT-activated condition, 30 cells in the P-activated and 28 in the T-activated conditions (results not shown). Judged by location, these populations included the three interneurons 157, 159, 162 that we studied in more detail and also several other interneurons previously identified as members of the local bend network (Lockery and Kristan, 1990b). However, since many additional cells were also classified as activated, we decided to present strictly restricted populations of clearly stimulus-activated cells in this study. In future studies, effects of statistical selection criteria should be compared across preparations to optimize the detection of stimulus-activated cells, which would lead to a more consistent picture of the network for tactile information processing.

The third reason for the incomplete activation maps could be the stimulation used in the experiments presented here. Even though the electrical stimulation of the P and/or T cell elicited spike trains mimicking typical responses to tactile skin stimulation (Pirschel and Kretzberg, 2016), the network received inputs from only one or two mechanoreceptors. In contrast, tactile stimulation always elicits responses of at least four mechanoreceptors, because each patch of the skin is covered by the overlapping receptive fields of two P cells and two T cells. For higher stimulus intensities, at least one N cell will react additionally. Since our VSD setup was limited to two intracellular electrodes, a complete simulation of the natural input to the tactile network by intracellular stimulation of four (or five) mechanoreceptors was not possible. Hence, if some interneurons are specifically tuned to relative temporal features of mechanoreceptor spike trains, e.g., coincidence detection they would not (or at least not optimally) respond to electrical stimulation of one P and one T cell, even though the timing of their spikes matches realistic skin stimulation. Hence, additional VSD experiments are needed with the skin attached to the ganglion to reveal a more complete network structure. Comparison of activity maps obtained for tactile stimulation to electrical stimulation of mechanoreceptor pairs or single mechanoreceptors can test our hypothesis of temporal processing on the level of interneurons.

Once these issues will be settled, combined electrophysiological, anatomical, and VSD studies applied to this small nervous system consisting of individually characterized cells can yield conclusive answers to fundamental questions of neural coding including the roles of spike counts versus spike timing, population coding and multiplexing. In particular, the analysis of combined encoding of multiple stimulus properties should be extended to a larger space of stimulus dimensions (e.g., velocity, shape, application angle, indentation depth, vibration, duration additionally to location and intensity). Moreover, the local bend response was reported to be modulated by feedback-loops in the network (Baljon and Wagenaar, 2015), by neuromodulators (Lockery and Kristan, 1991; Gaudry and Kristan, 2009), as well as by feeding status and environmental factors like water depth (Palmer et al., 2014). Hence, despite the low number of neurons involved in this seemingly so hard-wired network, the leech tactile system is also well suited for studies on general mechanisms underlying the flexibility of neural activity and behavior.

## Author contributions

All authors contributed to data analysis, interpretation of results, writing the manuscript and designing the figures. In addition, JK designed and coordinated the studies and drafted the text; FP performed intracellular recordings and skin stimulation, EF and GH performed VSD experiments, GH performed cell staining and confocal microscopy.

## Funding

Funding was provided by “Professorinnenprogramm” of Bundesministerium für Bildung und Forschung/Niedersächsisches Ministerium für Wissenschaft und Kultur (JK, GH, FP), by a fellowship of the graduate school “Neurosenses” of Niedersächsisches Ministerium für Wissenschaft und Kultur (FP), and by a fellowship of German Academic Exchange Service (EF).

### Conflict of interest statement

The authors declare that the research was conducted in the absence of any commercial or financial relationships that could be construed as a potential conflict of interest.
