# Supplementary material for: Encoding of Tactile Stimuli by Mechanoreceptors and Interneurons of the Medicinal Leech
Source: Front Physiol. 2016 Oct 28;7:506. doi: 10.3389/fphys.2016.00506 (PMC5083904; doi:10.3389/fphys.2016.00506)
Supplement: Supplementary file 2 [file DataSheet1.PDF]

Supplementary Material

Encoding of tactile stimuli by mechanoreceptors and interneurons of the medicinal leech

Jutta Kretzberg*, Friederice Pirschel, Elham Fathiazar, Gerrit Hilgen

*** Correspondence: Jutta Kretzberg: jutta.kretzberg@uni-oldenburg.de**

1 Supplementary Data: Linear Regression of T cell responses

For comparison of T cell on and off responses to different combinations of tactile stimulus intensity and location, the stimulus response curves presented in Figure 4D,E in the main manuscript were fitted by linear regression.

Corres-ponding Figure in main text	Cell response	Inten-sity [mN]	Spike count (suppl. Figure 1A,C)			Latency (suppl. Figure 1B,D)		
			Slope [sp/°]	y-inter-cept [sp]	Mean error [sp]	Slope [ms/°]	y-inter-cept [ms]	Mean error [ms]
4D	T on	10	-0.037	3.765	0.981	0.149	10.668	3.064
	T on	20	-0.033	4.249	0.912	0.121	9.444	2.561
	T on	50	-0.051	5.732	1.300	0.103	8.886	2.595
	T on	100	-0.059	8.190	2.062	0.104	8.537	2.706
4 E	T off	10	-0.007	1.157	0.400	0.329	4.005	16.030
	T off	20	-0.010	1.876	0.640	0.162	3.808	3.736
	T off	50	-0.030	3.398	1.369	0.142	3.141	3.748
	T off	100	-0.114	8.286	3.361	0.152	4.034	6.653

Supplementary Table 1. Parameters and obtained fitting errors for linear regressions of T cell responses, presented in Figure 4D,E in the main manuscript and in supplementary Figure 1.

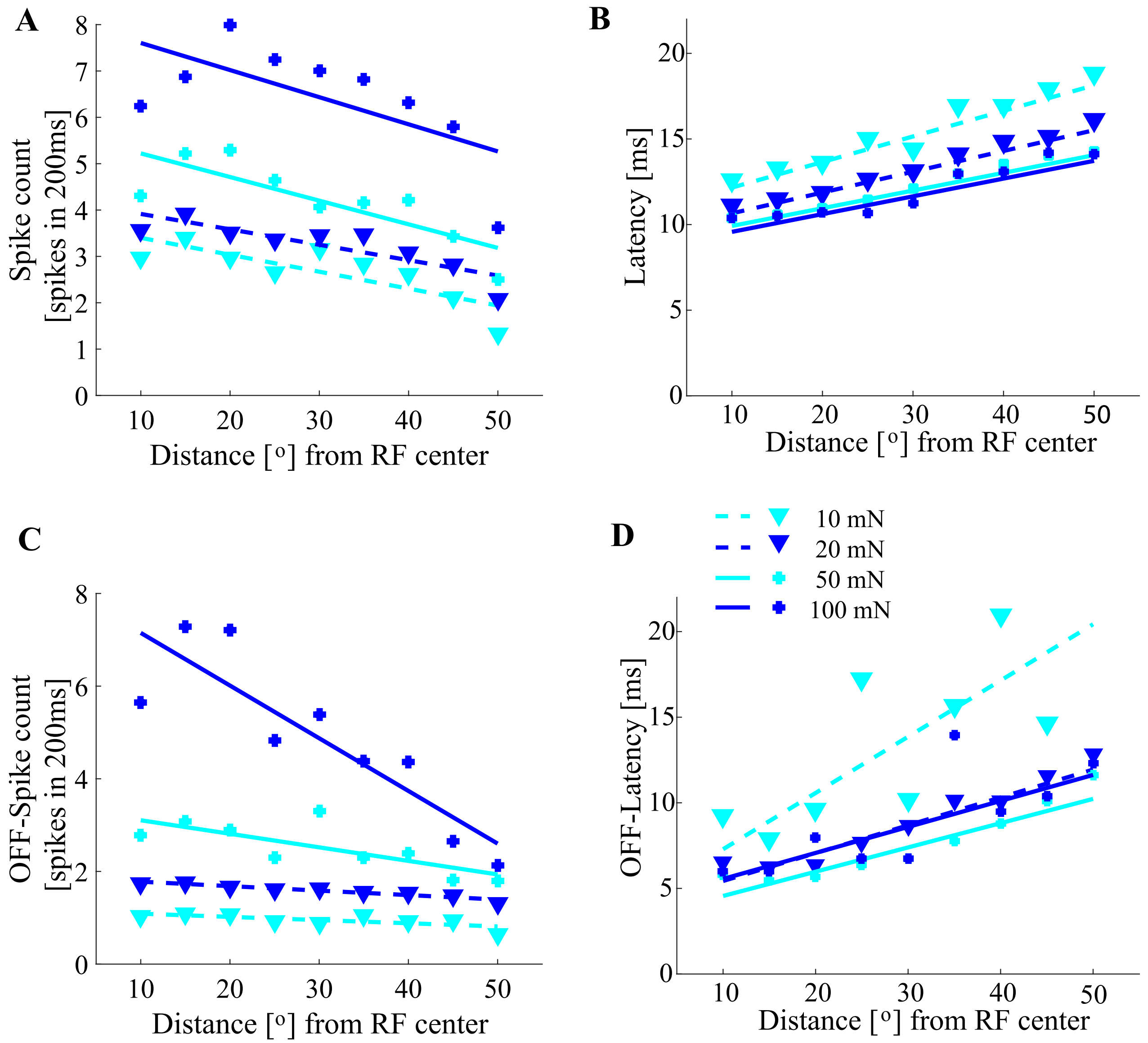

Supplementary Figure 2. Linear regression of the dependencies of T cell on and off responses to varied stimulus location for four different stimulus intensities. See supplementary Table 1 for fitting parameters and obtained fitting errors. Regression was applied to the data presented in Figure 4 of the main manuscript: Linear regression of the dependency of **(A)** spike count at stimulus onset, corresponding to Figure 4D left panel, **(B)** latency at stimulus onset, corresponding to Figure 4D left panel, **(C)** spike count of off responses, corresponding to Figure 4E left panel, **(D)** latency of off responses, corresponding to Figure 4D left panel, of T_v cells on stimulus location. For more details see legend of Figure 4 in main manuscript.

2 Supplementary Data: Animation of confocal image stack

Supplementary video 1. The morphological connections of T cells and interneurons 157 and 162 presented in Figure 6B in the main manuscript were detected based on a confocal image stack. Supplementary video 1 shows an animation of this stack. For experimental data see Methods section and legend of Figure 6 in the main manuscript.
